# Comparative pharmacology of a new recombinant FSH expressed by a human cell line

**DOI:** 10.1530/EC-17-0067

**Published:** 2017-04-27

**Authors:** Wolfgang Koechling, Daniel Plaksin, Glenn E Croston, Janni V Jeppesen, Kirsten T Macklon, Claus Yding Andersen

**Affiliations:** 1 Ferring Pharmaceuticals A/SCopenhagen, Denmark; 2 Bio-Technology General Israel LtdFerring Pharmaceuticals, Kiryat Malachi, Israel; 3 Croston ConsultingSan Diego, California, USA; 4 The Laboratory of Reproductive BiologyThe Department of Fertility at The Juliane Marie Centre, Rigshospitalet, Copenhagen University Hospital and The University of Copenhagen, Copenhagen, Denmark

**Keywords:** follicle-stimulating hormone, follitropin delta, gonadotropin clearance, recombinant FSH, FSH glycosylation

## Abstract

Recombinant FSH proteins are important therapeutic agents for the treatment of infertility, including follitropin alfa expressed in Chinese Hamster Ovary (CHO) cells and, more recently, follitropin delta expressed in the human cell line PER.C6. These recombinant FSH proteins have distinct glycosylation, and have distinct pharmacokinetic and pharmacodynamic profiles in women. Comparative experiments demonstrated that follitropin delta and follitropin alfa displayed the same *in vitro* potency at the human FSH receptor, but varied in their pharmacokinetics in mouse and rat. While follitropin delta clearance from serum depended in part on the hepatic asialoglycoprotein receptor (ASGPR), follitropin alfa clearance was unaffected by ASGPR inhibition in rat or genetic ablation in mice. The distinct properties of follitropin delta and follitropin alfa are likely to contribute to the differing pharmacokinetic and pharmacodynamic profiles observed in women and to influence their efficacy in therapeutic protocols for the treatment of infertility.

## Introduction

Follicle-stimulating hormone (FSH) released from the anterior pituitary in response to gonadotropin-releasing hormone (GnRH) plays a central role in reproduction in women, driving the growth and maturation of ovarian follicles, and regulating ovarian steroidogenesis (1). In addition, exogenous FSH plays a central role in the treatment of infertility (2), including *in vitro* fertilization (IVF) or intracytoplasmic sperm injection (ICSI), with the GnRH antagonist protocol increasingly common due to safety and convenience (3). As a result, continued innovation in infertility therapy is likely to involve both novel FSH proteins and novel treatment protocols.

FSH is composed of two extensively glycosylated protein subunits, and to express recombinant FSH (rFSH) for therapeutic use, the genes encoding the human FSH subunits are introduced into a mammalian cell line from which FSH protein is secreted and purified (4). Early recombinant FSH proteins expressed in Chinese Hamster Ovary (CHO) cells are termed follitropin alfa or follitropin beta (4, 5, 6), and display distinct glycosylation compared to urinary-derived endogenous human FSH, occurring as a range of isoforms of varying acidity that are on average slightly less acidic in isoelectric focusing (7). Compared to human urinary FSH, CHO cell-derived rFSH lacks bisecting GlcNAc residues, has a lower percentage of alpha 1–6 fucose and lacks alpha 2,6-linked sialic acid, while urinary FSH contains both alpha 2,3- and alpha 2,6-linked sialic acid (7, 8, 9).

Recently follitropin delta has been described (FE 999049), the first recombinant FSH protein in clinical development that is expressed in a human cell line (PER.C6), with individualized dosing optimized based on each patient’s weight and anti-Mullerian hormone (AMH) level (10, 11, 12). In female volunteers, follitropin delta displayed distinct pharmacokinetics and pharmacodynamics, with higher exposure and lower serum clearance than follitropin alfa (13). Moreover, the outcome of this phase 1 study demonstrated that the bioactivity of follitropin delta in the rat Steelman–Pohley assay compared to a reference standard did not directly predict proportional pharmacodynamic activity in women (13). These observations suggest that improved understanding of the distinct pharmacodynamics and pharmacokinetics of these recombinant FSH proteins will aid in their therapeutic application.

While follitropin alfa and follitropin delta have the same amino acid FSH sequence, they vary in their glycosylation (14). Follitropin delta has a higher proportion of tri- and tetra-sialylated glycans, with both alpha2,3- and alpha2,6-linked sialic acid, while follitropin alfa has only alpha2,3-linked sialic acid, in addition to other differences in glycosylation. More acidic isoforms of FSH with greater sialic acid modification and a lower isoelectric point are less potent *in vitro* at the human FSH receptor compared to more basic FSH isoforms, and also display lower serum clearance and longer circulating half-life (15, 16, 17, 18, 19).

To examine how the distinct glycosylation of follitropin alfa and follitropin delta affects their pharmacological activity, the *in vitro* and *in vivo* bioactivity of both recombinant FSH preparations was directly compared. For this purpose, the binding affinities of follitropin delta and follitropin alfa at the human FSH receptor were determined, and their bioactivities were tested in a HEK-293 cell line stably expressing the human FSH receptor, and in human granulosa cells isolated during oocyte pick-up prior to IVF. The pharmacokinetic behavior of the two rFSH proteins was also analyzed in rodents, examining the role of the ASGP receptor in mouse and rat on rFSH clearance from circulation.

## Methods

### Follitropin alfa and follitropin delta

Follitropin alfa produced in CHO cells was obtained from the market (Gonal F, Merck Serono, Batches BA018199A, AU009535, AU003768, BAD 04143, PS-3577, Y02A9111), and diluted and tested as indicated in each experimental method. Follitropin delta samples produced in the human cell line PER.C6 were provided by Ferring Pharmaceuticals (Batches FSMB01/2, G16641, G11105, CE0041, 1178-160) and characterized and diluted for testing as indicated in each experimental procedure. The first international standard for recombinant FSH (NIBSC 92/642) was obtained from NIBSC (National Institute for Biological Standards and Control).

### Human FSH receptor binding

The binding displacement assay for the human FSH receptor was performed utilizing [Propionyl-^3^H] FSH as the radiolabel (Quotient Research, UK). Incubation Buffer and Stop Buffer was 10 mM Tris, 25 mM MgCl_2_, 0.5% BSA, pH 7.5. Follitropin delta used in these experiments had a protein concentration of 0.62 mg/mL and an assumed molecular weight of 33,000 g/mol. A sample of follitropin alfa with an assumed molecular weight of 31,000 g/mol, with 5.5 µg or 75 IU powder dissolved in 1.2 mL of the incubation buffer and the stock solution at 0.15 µM was aliquoted and stocked at −20°C. For both compounds, all dilutions were performed in the incubation buffer.

To make membranes, a frozen pellet of HEK-293 cells expressing human FSH receptor (approximately 600 million cells) was suspended in buffer containing 50 mM Tris–HCl, 5 mM EDTA–Tris, 20 mM NaCl, 5 mM KCl, 5 mM MgCl_2_, 1.5 mM CaCl_2_, 10 μg/mL trypsin inhibitor, 1 μg/mL leupeptin, 75 μg/mL PMSF, pH 7.4. After centrifugation at 50,000 ***g*** for 15 min (4°C), the pellet was suspended in the same buffer with the addition of 10% glycerol. The protein concentration was determined with the Bradford methodology and the aliquots was stored at −80°C.

To initiate binding, FSH receptor membranes containing 180 µg protein were incubated one hour at 37°C with 0.4 nM of [Propionyl-^3^H] FSH and the indicated quantity of follitropin alfa or follitropin delta. Unifilter GF/B filters (Whatman, UK) were pre-soaked with 10 mM Tris–HCl, 25 mM MgCl_2_, 0.5% BSA, pH 7.5. Incubated mixtures were filtered through pre-soaked filters using a Packard FilterMate Harvester, and washed 3–4 times using the same buffer. Filters were then dried, MICROSCINT 0 scintillation fluid (PerkinElmer) was added (30 µL/well) and scintillation counting was performed using a Packard Topcount NXT instrument (1 min per well).

*K*_i_ was estimated by the Cheng–Prusoff equation (20).

### *In vitro* bioactivity using a HEK-293 cell line expressing the human FSH receptor

Human embryonic kidney 293 (HEK-293) cells stably transfected with the human FSH receptor (21) were thawed from frozen aliquots, and grown and passaged in DMEM with 4.5 g/L d-glucose (Biological Industries, Israel) containing 5% fetal bovine serum, heat inactivated (Biological Industries) and 2 mM l-glutamine (Biological Industries). For assays, hFSH receptor expressing cells were incubated with the indicated concentrations of rFSH proteins for 90 min. At the end of the incubation, cells were lysed and cAMP produced in response to rFSH stimulation quantitated using the DiscoveRx HitHunter cAMP XS + cAMP assay kit (DiscoveRx, Fremont, CA, USA), measuring luminescence using the TECAN Infinite F200 plate reader and reporting RLU. Data were analyzed using four-parameter curve-fitting (Graphpad Prism).

### *In vitro* bioactivity using fresh luteinized granulosa cells from IVF patients

To determine the activity of rFSH proteins, human granulosa cells were collected from 13 women ages 26–39 years (34.5 ± 1.2 years, mean ± s.e.m.) undergoing IVF treatment at the University Hospital of Copenhagen, with approval of the project by the ethical committee of the municipalities of Copenhagen and Frederiksberg (H-32013-201). The studies in which rFSH proteins were compared utilized cells from eight of these patients. Women were either stimulated following a long agonist protocol or a standard antagonist protocol. In all cases, a bolus injection of hCG was used for final oocyte maturation 36 h prior to oocyte collection. Granulosa cells were collected in connection with aspiration of oocytes for IVF treatment. After oocytes were removed from the follicular aspirates, the granulosa cells from each patient were collected in a pool and purified using a lymphoprep gradient (Stemcell Technologies, Grenoble, France) where fluid was maintained in the upper layer, while red blood cells were collected in the pellet and the granulosa cells were located in the intermediate layer. After aspiration of the granulosa cells layer, large clumps of cells were removed under a microscope and stored at −80°C until used for DNA purification and detection of the 307/680 FSHR and the −29 FSHR polymorphism of the patient (22). A single cell suspension of granulosa cells was prepared from the smaller clumps of cells by the addition of the proteolytic enzyme mix Tryple (mainly trypsin) for up to 5 min or until the clumps became disintegrated. Proteases were inactivated by the addition of FBS, and the suspension was centrifuged (400 ***g*** 10 min) to pellet the granulosa cells. Granulosa cells were cultured in 4-well dishes (Nunc, Roskilde, Denmark) at 37°C with 6.5% O_2_ in culture medium consisting of MEM Alpha medium (Gibco) supplemented with human serum albumin 10 mg/mL (CSL Behring, Lyngby, Denmark); 50 mg/mL FBS (Gibco); 2 mM GlutaMAX (Gibco), Insulin–Tranferrin–Selenium mix (Gibco); and Pen/strep (Gibco). The first 24 h cells were allowed to attach to the bottom in the presence of 50 mg/mL FBS after which the culture medium was changed. After 48 h, cells were stimulated by rFSH for 24 h. Thereafter the media was harvested, snap frozen and stored at −80°C until thawed for hormone measurements. The cells attached to the bottom were washed in pre-warmed PBS (Gibco) prior to being lysed. The granulosa cells were lysed with the lysis buffer purchased with the mRNA purification kit (see below), snap frozen and stored at −80°C until RNA purification and qPCR.

Purification of mRNA was performed using Agilent Absolutely RNA nanoprep kit (Agilent Technologies). All steps were performed on ice, apart from the elution of isolated mRNA. First strand cDNA synthesis was performed using Applied Biosystems High Capacity cDNA reverse transcription kit (Applied Biosystems) with following temperature profiles: 25°C for 10 min, 37°C for 120 min, 85°C in 5 s and 4°C until the synthesis was terminated. All first-strand cDNA syntheses were performed on a Thermal cycler (ThermoFisher Scientific Arktik thermal cycler block). TaqMan Universal PCR master mix (Applied Biosystems) and pre-designed TaqMan probes were tagged with a FAM-labeling (Applied Biosystems): *Cyp19a1* (Hs00903412_m1), *3beta-HSD* (Hs01084547_gH), *INHA* (HS00171410_m1) and *GAPDH* (Hs99999905_m1) as a housekeeping gene. qPCR reactions were performed in a total volume of 10 µL consisting of a mixture of 0.5 µL 20× TaqMan gene expression assay, 5.0 µL 2× TaqMan gene expression master mix, 2.0 µL cDNA and 2.5 µL RNase free water for each single reaction. The qPCR plates (Roche Diagnostics) were centrifuged at 1000 ***g*** before analysis using the LightCycler 480 (Roche) with the following program for 45 cycles; pre-incubation: 50°C for 2 min, followed by 95°C for 10 min, amplification at 95°C for 15 s and 60°C for 1 min, followed by a quantification measurement, ending by cooling to 40°C in 30 s. All samples were run in duplicates and normalized to the corresponding *GAPDH* gene expression value.

The calculation of the expression level of each individual gene was carried out according to the Comparative CT Method for relative quantification of gene expression (23). Concentrations of estradiol and progesterone were measured using commercially available ELISA kits (NovaTec Immundiagnostica, Germany). A PBS solution containing 1% BSA was used for dilution of the culture media samples prior to measurements. Dilutions of 1:10 for estradiol and 1:500 of progesterone secured readings inside of the standard curve. All samples were run in duplicate, and the mean of the replicates was used. Inhibin A and inhibin B were measured using commercially available ELISA kits (AnshLabs, Webster, USA). Serum from post-menopausal women devoid of inhibin A and inhibin B was used to dilute samples.

### Steelman–Pohley assay

Follitropin alfa was obtained from a freeze-dried vial stated by the manufacturer to contain 600 IU of rFSH per and filled by mass to contain 44 µg of FSH per mL. Follitropin delta was measured by size exclusion chromatography HPLC to contain 39 µg of FSH per mL and 600 IU/mL in liquid formulation.

The Steelman–Pohley assay (24) was performed according to *USP* monograph for Menotropins and the *Ph Eur* monograph for Urofollitropin (USP Monograph: Mentotropins 4/30/08, PhEUR: Urofollitropin 01/2008/0958), with immature female Sprague–Dawley rats (Harlan, Jerusalem, Israel), 20–21 days old (35–45 g) having weights such that the difference between the heaviest and the lightest rat is not more than 10 g. Animals were fed a standard diet and allowed free access to water, randomly distributing animals for the experiment with seven animals in each group.

Solution A used for the dilution of proteins for injection was 130 mM NaCl, 75 mM Na_2_HPO_4_–2H_2_O, 0.1% BSA, pH 7.5. hCG was diluted to 700 IU/mL in Solution B, which includes 130 mM NaCl, 75 mM Na_2_HPO_4_–2H_2_O, 1% BSA, pH 7.5, and further diluted in Solution A to 70 IU/mL, which was then used for the preparation of proteins and controls for injection.

For each rFSH protein, a 0.2 mL volume was injected once per day for three days at three dose levels subcutaneously in the dorsal area for each, according to established assay guidelines, with the injection also containing 14 IU of hCG (Serono). The weight of each animal was recorded at the beginning and end of each experiment. Twenty-four hours after the last injection, animals were killed, and ovaries were removed and cleaned of extraneous tissues, blotted dry, and pairs of ovaries weighed.

### Specialized rat pharmacokinetics (ASF competition)

Identical volumes (0.5 mL) and concentrations (4 µg/mL) of follitropin delta and follitropin alfa were injected IV into male Sprague–Dawley rats weighing 80–85 g (Harlan) in the presence and absence of asialofetuin (Sigma). Animals (2 per treatment group) were bled at specific time points (0.25, 1, 2 and 4 h post injection), and sera were tested for FSH concentration by ELISA in duplicates (DRG Instruments GmbH, Marburg, Germany). For the ELISA, 100 µL of anti-FSH enzyme conjugate was added to samples, and plates were incubated for 30 min at room temperature. Contents of wells were removed and rinsed 5 times with water, inverting plates to dry. Substrate solution was added and incubated 5 min at room temperature before adding 50 µL of stop solution, and absorbance was read at 450 nM. The anti-human FSH antibody used in the ELISA for the detection of recombinant human FSH proteins displayed no measureable interference from rat or mouse serum (data not shown). Results in the presence and absence of ASF were compared using a Student’s *t-*test at *P* < 0.05.

### Specialized mouse pharmacokinetics (*Asgpr* knockout model)

Homozygous asialoglycoprotein receptor knockout (KO) mice (B6;129S7-Asgr2tm1Her/J) (25) were obtained from The Jackson Laboratory, and wild-type C57Bl/6J mice were obtained from Harlan Laboratories. Weight of the mice was from 22 to 24 g. Follitropin alfa freeze-dried powder was reconstituted in the laboratory with dissolving solution supplied. Follitropin delta drug product was received in a solution form. Identical volumes (0.5 mL) and concentrations (6 µg/mL) of follitropin alfa and follitropin delta were injected subcutaneously (SC) into both male C57Bl/6J mice and male *Asgpr* knockout mice. Animals (*n* = 3 per treatment group) were bled at specific time points thereafter (0.5, 3, 6, 9 and 24 h post dosing), and sera were tested for FSH concentration by ELISA in triplicates.

The mean serum concentrations of follitropin delta were normalized based on the ELISA concentration of the original injected vials compared to follitropin alfa. Serum FSH concentration vs time was plotted, and a classical trapezoidal rule was used to compute the area under the curve (AUC) by ‘PK Solutions 2.0’ software. The AUC value of FE follitropin delta is presented as a percent of the AUC value calculated for follitropin alfa. Results at each time point were compared between wild-type and *Asgpr* knockout animals using a Student’s *t-*test with *P* < 0.05 as the criteria for significance.

## Results

### Human FSH receptor binding

**Figure 1:**
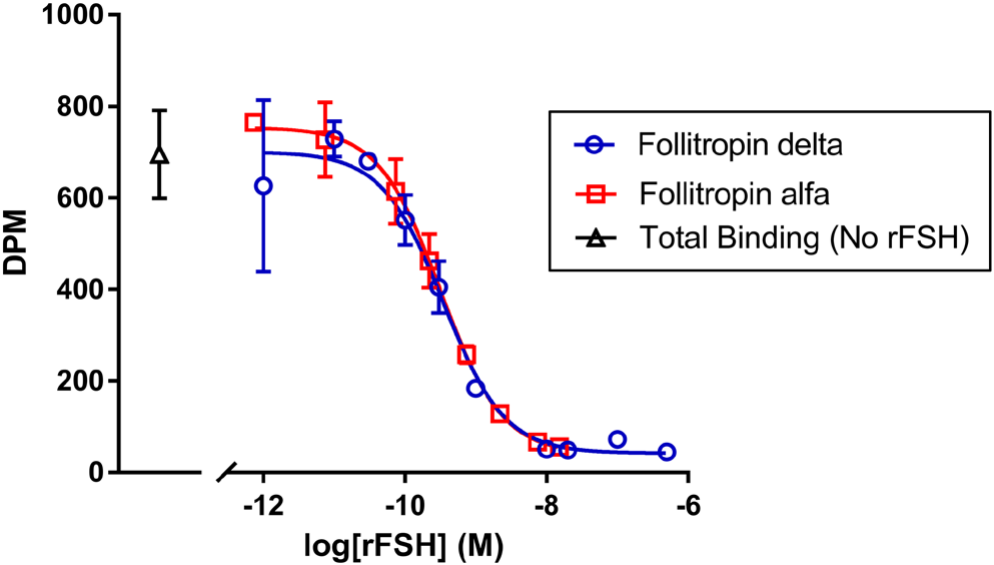
The displacement of radiolabeled propionyl-^3^H FSH from human FSH receptor was measured as a reduction in dpm bound across the indicated concentrations of follitropin delta (open circles, blue) and follitropin alfa (open squares, red), or total binding in the absence of unlabeled recombinant FSH protein (open triangle, black). Each concentration was tested in duplicate, with the mean and s.d. indicated. Data were fit using four-parameter curve-fitting. The results for one experiment are shown, whereas results from two other tests producing similar results are not shown.

FSH receptor binding affinity was measured by displacement of radiolabeled FSH from the human FSH receptor. Propionyl-^3^H labeled FSH was used as the radiolabeled ligand in these studies, with membranes prepared from recombinant HEK 293 cells expressing the human FSH receptor. The *K*_d_ value of [Propionyl-^3^H] FSH in this assay was 0.2 nM with a *B*_max_ of 28 fmol/mg protein (data not shown). The average IC_50_ in three experiments for displacement of propionyl-^3^H FSH was virtually identical for the two rFSH proteins, 0.302 nM for follitropin alfa and 0.299 nM for follitropin delta, with the results from a representative experiment shown in Fig. 1, resulting in a calculated *K*_i_ of approximately 0.1 nM for both proteins.

### *In vitro* bioactivity using a HEK-293 cell line expressing the human FSH receptor

**Figure 2:**
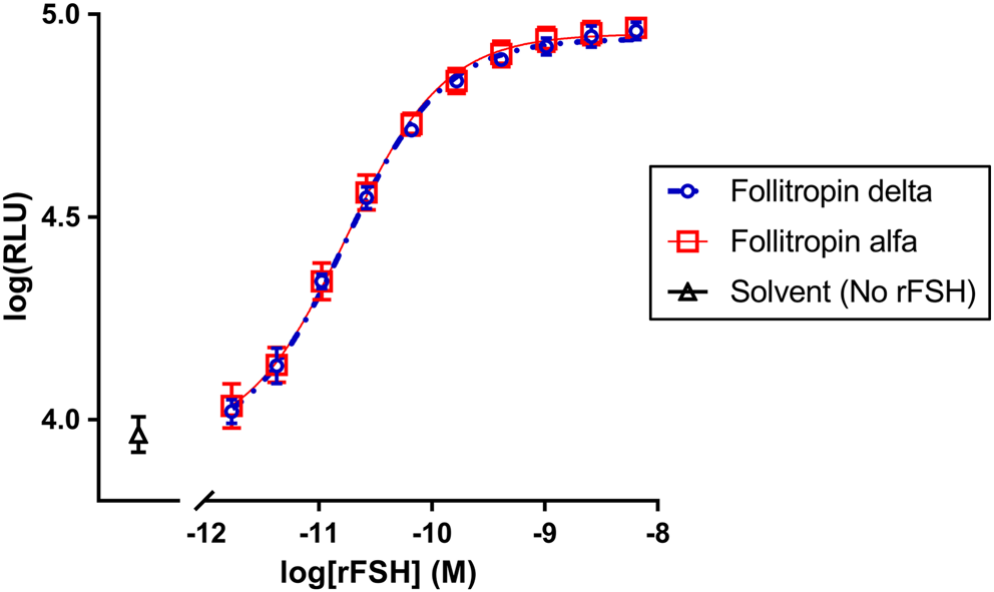
Production of cAMP in response to activation of the human FSH receptor in stably transfected HEK-293 cells was measured across the indicated concentrations of follitropin delta (open circles, blue), follitropin alfa (open squares, red) or no recombinant FSH (open triangles, black) with increasing luminescence (RLU) proportional to increasing cAMP present. Each concentration of rFSH proteins was tested in quadruplicate, with the mean and s.d. shown.

To compare the *in vitro* bioactivity of follitropin delta and follitropin alfa, both rFSH proteins were incubated *in vitro* with human embryonic kidney cells (HEK-293 cells) engineered to stably express the human FSH receptor, measuring production of the second messenger cAMP. The potency of the two rFSH proteins was virtually identical in this assay (Fig. 2), with follitropin alfa activating the hFSH receptor with an EC_50_ of 0.0174 nM, and follitropin delta producing an EC_50_ of 0.0171 nM. In a second independent experiment, follitropin alfa produced an EC_50_ of 0.0182 nM and follitropin delta produced an EC_50_ of 0.0181 nM (data not shown).

### *In vitro* bioactivity using fresh luteinized granulosa cells from IVF patients

**Figure 3:**
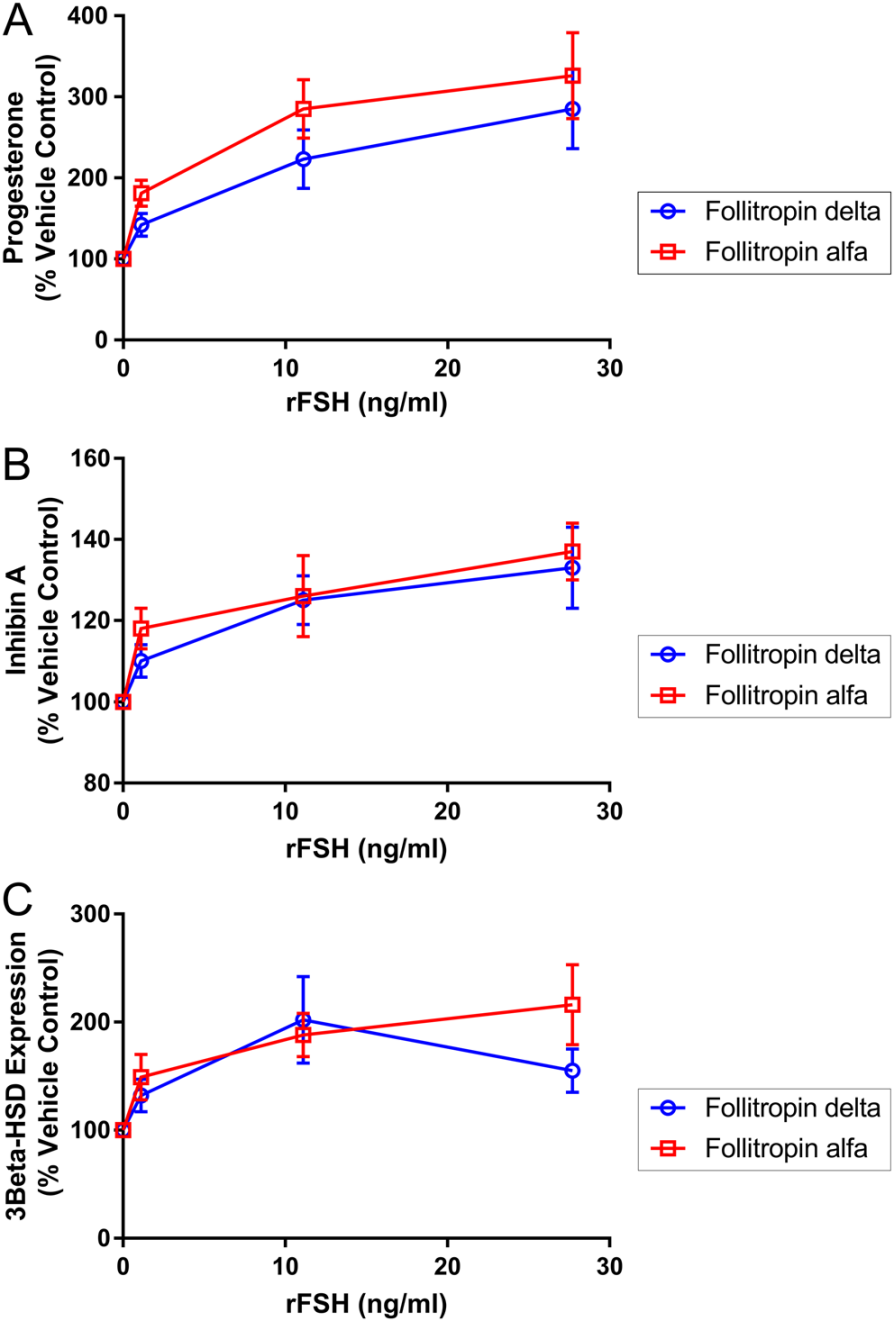
The induction of hormone release and increase in gene expression were measured in human granulosa cells exposed to rFSH proteins *in vitro*. Cells from eight patients were included, with duplicate samples of cells from each patient tested at each rFSH concentration, normalizing responses relative to the level observed in the absence of rFSH (100%), with data points reflecting the mean and s.e.m. The response for follitropin delta (open circles, blue) and follitropin alfa (open squares, red) is shown in terms of progesterone secretion (A), inhibin A secretion (B) and *3beta-HSD* gene expression (C).

The *in vitro* bioactivity of follitropin delta and follitropin alfa was then compared using luteinized granulosa cells isolated from follicles of IVF patients and expressing the endogenous human FSH receptor. These experiments examined the impact of rFSH proteins on granulosa cell production of estradiol, progesterone, inhibin A and inhibin B, and on the expression of genes including *CYP19a1*, *3B-HSD* and *Inh A*. Granulosa cells were collected following triggering of final follicle maturation at oocyte pickup from a total of 13 women undergoing controlled ovarian stimulation prior to IVF. Both rFSH proteins induced progesterone production approximately 300% normalized to basal levels in the absence of rFSH (*P* > 0.1), and with similar potency (Fig. 3A). Inhibin A in supernatant was increased by both rFSH proteins approximately 35–40% over basal (Fig. 3B), while estrogen and inhibin B were not significantly increased by either rFSH protein (data not shown). The expression of FSH regulated genes was studied in the same human granulosa cell studies, preparing RNA at the end of the incubation period to be quantitated. Expression of *3B-HSD* was induced approximately 200% by both rFSH proteins (Fig. 3C), the *CYP19a1* gene was induced 40–50% by both rFSH proteins (data not shown) and *INHA* expression was induced by both rFSH proteins 50–100% over basal levels (data not shown).

### Steelman–Pohley assay

**Figure 4:**
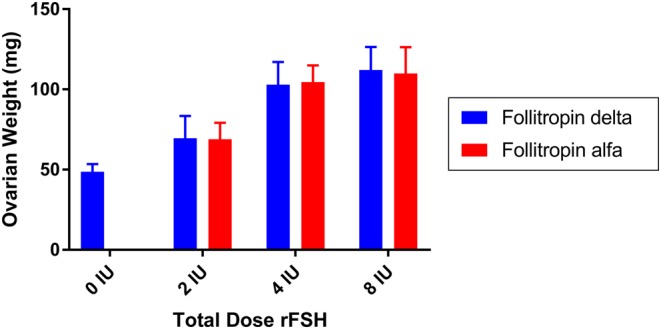
The bioactivity of follitropin delta (blue bars) and follitropin alfa (red bars) was compared *in vivo* in rat in the Steelman–Pohley bioassay, measuring the increase in ovarian weight with the administration of the indicated total dose of rFSH proteins. Bars are the mean of 14 animals in each dose group, and error bars are the s.d.

The Steelman–Pohley bioassay concept was used to demonstrate the bioactivity of follitropin delta in rat compared to an international reference standard of recombinant FSH produced in CHO cells. When follitropin delta and follitropin alfa were compared by Steelman–Pohley assay based on dosing of a previously assessed bioactivity, the two compounds produce parallel curves and, thus, are very similar in their pharmacodynamic behavior in rat (Fig. 4).

### Specialized rat pharmacokinetics (ASF competition)

**Figure 5:**
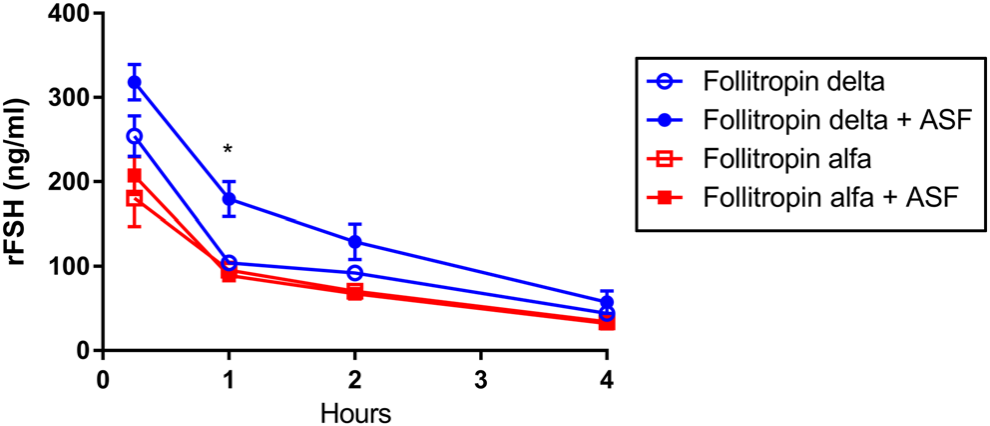
The clearance of follitropin delta (blue circles) and follitropin alfa (red squares) was compared following intravenous injection in rat, in the presence of a saturating dose of ASF (filled symbols), or the absence of co-injected ASF (open symbols). Serum taken at the indicated time points was analyzed for rFSH concentration. A Student’s *t*-test compared results in the presence or absence of ASF, with an asterisk (*) indicating a significant difference (*P* < 0.05).

To examine the role of the ASPGR in clearance of follitropin delta and follitropin alfa, the rFSH proteins were injected intravenously in rats in the presence or absence of a saturating dose of asialofetuin (ASF), a ligand for the ASGPR that can compete and block the hepatic clearance of other ASGPR ligands. Co-injection of a saturating dose of ASF reduced the clearance of follitropin delta from serum, resulting in a 42% increase in the AUC compared to the absence of ASF (Fig. 5) and a significant difference in ASF level in plasma at one hour while co-injection with ASF did not significantly affect follitropin alfa clearance or plasma levels.

### Specialized mouse pharmacokinetics (*Asgpr* knockout model)

**Figure 6:**
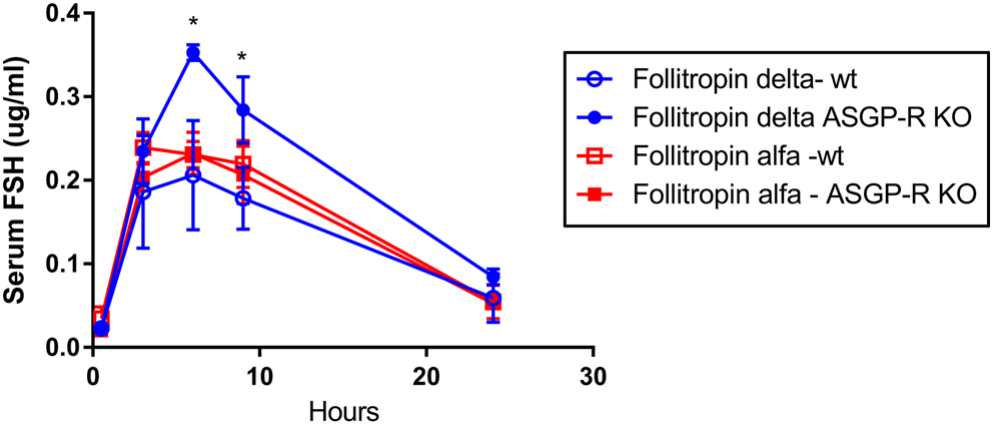
The clearance of follitropin delta (blue circles) and follitropin alfa (red squares) was compared in wild-type mice (open symbols) and *Asgpr* knockout mice (filled symbols), determining rFSH concentration present in serum at varying time points. A Student’s *t*-test compared results in the presence or absence of ASF, with an asterisk (*) indicating a significant difference (*P* < 0.05).

To further examine the role of ASGPR in clearance of follitropin alfa and follitropin delta, their pharmacokinetics were compared in wild-type and ASGPR knockout mice, injecting identical volumes and concentrations of protein subcutaneously (Fig. 6). Follitropin alfa and follitropin delta displayed similar pharmacokinetic profiles following subcutaneous injection in wild-type mice. In ASGPR deficient mice, however, follitropin delta clearance from serum was reduced, and the AUC in serum following follitropin delta injection increased 40% compared to follitropin alfa, with a significant difference in plasma concentrations at the six- and nine-hour time points, further supporting the hypothesis that follitropin delta clearance from serum involves the ASGPR while follitropin alfa clearance relies less on this mechanism.

## Discussion

Follitropin delta and follitropin alfa display distinct pharmacokinetic and pharmacodynamic properties in healthy female volunteers, with follitropin delta displaying lower clearance from serum, producing greater exposure and a greater pharmacodynamic response (13). In addition, follitropin delta displays a noninferior pregnancy rate and live birth rate compared to follitropin alfa, with a reduced rate of complications such as OHSS (39). The differences observed clinically between the two rFSH proteins might be caused by differences in their glycosylation (14) since variations in glycosylation have been reported to affect FSH pharmacokinetics and pharmacodynamics (15, 16, 17, 18, 19). To better understand the clinical differences observed between the two rFSH proteins, we compared their signaling through the human FSH receptor and their pharmacokinetics in rodents.

When compared directly it was found that follitropin delta and follitropin alfa elicit indistinguishable activity at the human FSH receptor *in vitro*. The two rFSH proteins displayed the same apparent binding affinity for the human FSH receptor, and virtually identical potency in the activation of the human FSH receptor in a HEK-293 cell-based assay, measuring the induction of cAMP production in response to agonist ligand. Differences in the clinical activity of the two rFSH proteins are unlikely to be caused by differences in their binding and activation of the FSH receptor.

Further comparing the two rFSH proteins, they produced similar *in vitro* responses in patient-derived luteinized granulosa cells, measuring FSH-induced hormone production and gene regulation. In addition to inducing progesterone production to similar levels, both rFSH proteins increased *3B-HSD* expression, the enzyme responsible for the conversion of pregnenolone to progesterone (26). The modest induction of *CYP19a1* gene expression by both rFSH proteins is consistent with previous reports in granulosa cells (27, 28, 29) as was the induction of both *INHA* gene expression and inhibin A protein secretion (30). The lack of increase in estrogen and inhibin B in these studies is consistent with the stage at which the granulosa cells were collected, after patients had been through controlled ovarian stimulation and triggering of final oocyte maturation, shifting them from follicular gene expression and hormone production toward luteal phase gene expression (31). Also, the absence of a source of steroidal precursors for estrogen biosynthesis in this *in vitro* system prevented significant estrogen production (32).

The differences in follitropin delta and follitropin alfa responses in rats and in women may be due in part to differences in pharmacokinetics, including clearance. A potential mechanism of rFSH clearance from serum is by hepatic asialoglycoprotein receptor (ASGPR), which mediates the rapid clearance from serum of glycoproteins containing terminal galactose, GalNac or alpha2,6 sialic acid, and GalNac with 2.6 sialic acid, the latter having a high affinity for the rat ASGPR (33, 34).

Inhibition of ASGPR-dependent clearance in rats by co-injection of a saturating dose of ASF (35) reduced clearance of follitropin delta from serum but did not affect follitropin alfa clearance. Similarly, the clearance of follitropin delta was reduced in *Asgpr* knockout mice compared to wild-type mice, further supporting the role of the hepatic ASGPR in clearance of follitropin delta but not follitropin alfa from serum. This difference in rFSH clearance from serum by the liver is likely to result in differing metabolic fates for the two rFSH proteins.

Other factors might also play a role in the distinct pharmacokinetics of follitropin delta. Human expression of hepatic ASGPR is lower than in mouse or rodent, suggesting less dependence on this clearance mechanism in humans (36). Renal clearance also plays a role in FSH pharmacokinetics, and the greater sialic acid content of follitropin delta compared to follitropin alfa, with increased charge and size, is likely to result in lower renal clearance (37, 38) as well as impacting hepatic clearance.

The distinct properties of follitropin delta have important implications for its pharmacology and clinical use. The Steelman–Pohley assay for the determination of FSH bioactivity utilizes a recombinant reference standard (1st IS FSH, recombinant 92/642) expressed in CHO cells. As a result, bioactivity in the Steelman–Pohley assay compared to the reference standard does not directly predict the pharmacodynamic response of follitropin delta in humans, as was observed. Dosing follitropin delta according to mass rather than bioactivity measured in rat is important as a result. This principle is being pursued in follitropin delta clinical development, with individualized dosing by mass optimized for each patient based on their AMH level and weight (11, 12). The differences between the pharmacology of follitropin delta and follitropin alfa also suggest that one should not be directly substituted for the other in one clinical procedure. Future innovation in the treatment of infertility should continue to consider the impact of the unique properties of rFSH proteins on their therapeutic use.

## Declaration of interest

Glenn Croston was paid as a consultant by Ferring Pharmaceuticals. Daniel Plaksin is an employee of BTG Israel, a subsidiary of Ferring Pharmaceuticals. Claus Yding Andersen, Janni V Jeppesen and Kirsten T Macklon received funding from Ferring Pharmaceuticals. Wolfgang Koechling is an employee of Ferring Pharmaceuticals A/S.

## Funding

This research was funded by Ferring Pharmaceuticals and its subsidiaries.
